# Calcium imaging of adult olfactory epithelium reveals amines as important odor class in fish

**DOI:** 10.1007/s00441-024-03859-w

**Published:** 2024-02-13

**Authors:** M. Dieris, D. Kowatschew, T. Hassenklöver, I. Manzini, S. I. Korsching

**Affiliations:** 1 Institute of Genetics, Faculty of Mathematics and Natural Sciences of the University at Cologne, Zülpicher Str. 47A, 50674 Cologne, Germany; 2 Institute of Neurophysiology and Cellular Biophysics, University of Göttingen, Göttingen Germany, and Center for Nanoscale Microscopy and Molecular Physiology of the Brain (CNMPB), Göttingen, Germany; 3 Current address: Department of Animal Physiology and Molecular Biomedicine, Institute of Animal Physiology, Justus-Liebig-University Gießen, Gießen, Germany

**Keywords:** Olfaction, Odorant, Zebrafish, Amine, Ciliated neuron

## Abstract

The odor space of aquatic organisms is by necessity quite different from that of air-breathing animals. The recognized odor classes in teleost fish include amino acids, bile acids, reproductive hormones, nucleotides, and a limited number of polyamines. Conversely, a significant portion of the fish olfactory receptor repertoire is composed of trace amine-associated receptors, generally assumed to be responsible for detecting amines. Zebrafish possess over one hundred of these receptors, but the responses of olfactory sensory neurons to amines have not been known so far. Here we examined odor responses of zebrafish olfactory epithelial explants at the cellular level, employing calcium imaging. We report that amines elicit strong responses in olfactory sensory neurons, with a time course characteristically different from that of ATP-responsive (basal) cells. A quantitative analysis of the laminar height distribution shows amine-responsive cells undistinguishable from ciliated neurons positive for olfactory marker protein. This distribution is significantly different from those measured for microvillous neurons positive for transient receptor potential channel 2 and basal cells positive for proliferating cell nuclear antigen. Our results suggest amines as an important odor class for teleost fish.

**Supplementary Information:**

The online version contains supplementary material available at 10.1007/s00441-024-03859-w.

## Introduction

The vertebrate sense of smell has emerged in aquatic organisms but is much better studied in terrestrial, in particular mammalian species (Mombaerts 2004, 2006). The basic principles of olfactory information processing appear to be the same in terrestrial and aquatic vertebrates, such as monogenic expression of olfactory receptors, nearly random expression of olfactory receptors in the sensory surface, convergence of the axons of same receptor-expressing sensory neurons into one to two glomeruli in the olfactory bulb, and some degree of chemotopy in the olfactory bulb (Mombaerts 2004, 2006; Korsching 2020). However, aquatic organisms rely on their odorants being water-soluble, while terrestrial organisms require volatile odorants, which by necessity should have less strong interactions with the solvent. Thus one expects odorants of aquatic vertebrates to be quite different from those important for terrestrial vertebrates. In fact, large molecules such as steroids and prostaglandins are odorless for humans, but constitute important odors for fish, serving as reproductive pheromones (Stacey et al. 2003). The (few) olfactory receptors found for this class of molecules belong to the odorant receptor family (ORs; Yabuki et al. 2016).

Moreover, charged molecules such as amino acids and nucleotides, which are mostly odorless for humans, constitute an important class of odorants for fish, serving as indicator of nutrient sources (Hara 1975; Friedrich and Korsching 1997, 1998). Amino acid detection is likely mediated by a large group of class C GPCRs, the V2Rs, also named OlfC in teleost fish (DeMaria et al. 2013). Concerning nucleotide detection, current knowledge points to a single olfactory receptor, adorb (Kowatschew et al. 2022), which is highly specific for adenosine, generated within the olfactory mucosa through the hydrolysis of ATP (Wakisaka et al. (2017). Recently, in addition to sparse olfactory sensory neuron (OSN) responses to ATP, non-olfactory responses to ATP have been shown in supporting and basal cells of zebrafish (Demirler et al. 2020), similar to earlier observations made in larval *Xenopus* (Czesnik et al. 2006).

Amines constitute a bimodal odor class which can function in both aquatic and terrestrial environments. In mammals they are detected by a small family of trace amine-associated receptors (TAARs; Liberles and Buck 2006). The orthologous family is much expanded in teleost fish and heterologous expression experiments have identified many amines as ligands (Hussain et al. 2013; Li et al. 2015; Sharma et al. 2016). This would suggest amines as a major odor class in fish. However, electroolfactogram studies, which measure local field potentials, have shown responses only to few amines, such as spermidine and cadaverine (Rolen et al. 2003; Michel et al. 2003), and behavioral responses have been shown for even less amines (Hussain et al. 2013).

Here we used calcium imaging with cellular resolution to identify amine responses in slice preparations of zebrafish olfactory epithelium. The response characteristics are significantly different from those obtained for ATP which is known to activate mostly nonneuronal cells (Demirler et al. 2020). The spatial pattern of amine-responding cells is undistinguishable from that of ciliated neurons and different from that of microvillous neurons and basal cells. Our results suggest amines as an important odor class for teleost fish.

## Results

### Imaging odor responses in slice preparations of zebrafish olfactory epithelium

**Fig. 1:**
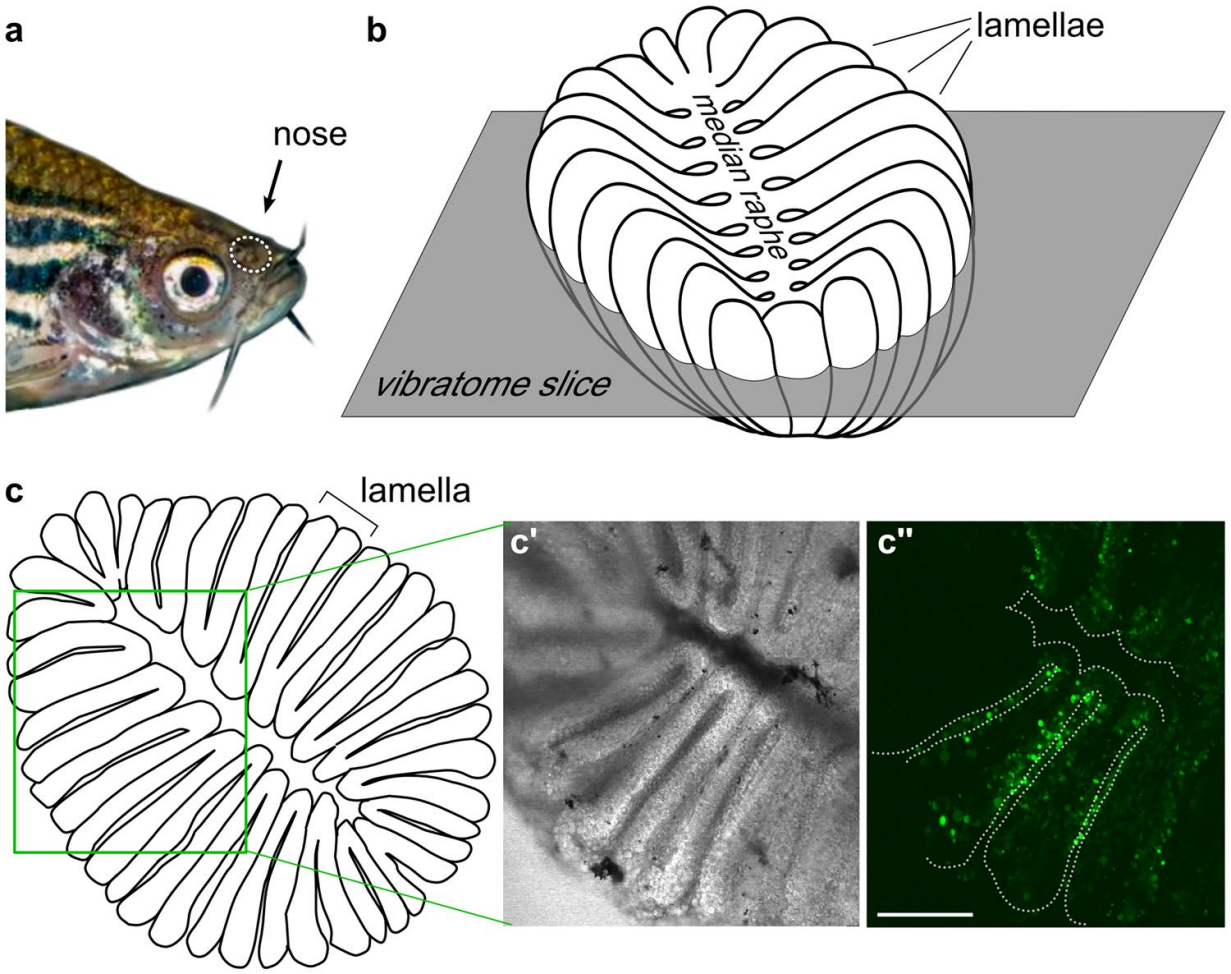
Slice preparation of adult zebrafish olfactory epithelium for calcium imaging. The position of the nose is shown by the white dotted oval (**a**). Schematic representation of the olfactory epithelium (**b**), lumen is up, individual lamellae and the median raphe are visible. The horizontal orientation and approximate height of the vibratome section plane is shown. *En face* view of the vibratome section (**c**), cross-sectioned lamellae and the median raphe are visible. In the schematic representation (**c**), the green rectangle corresponds approximately to the region shown in (**c'**, **c''**). A representative vibratome section used for calcium imaging (bright field (**c**') and fluorescent signals (**c''**) after loading with Fluo4-AM, respectively)

**Fig. 2:**
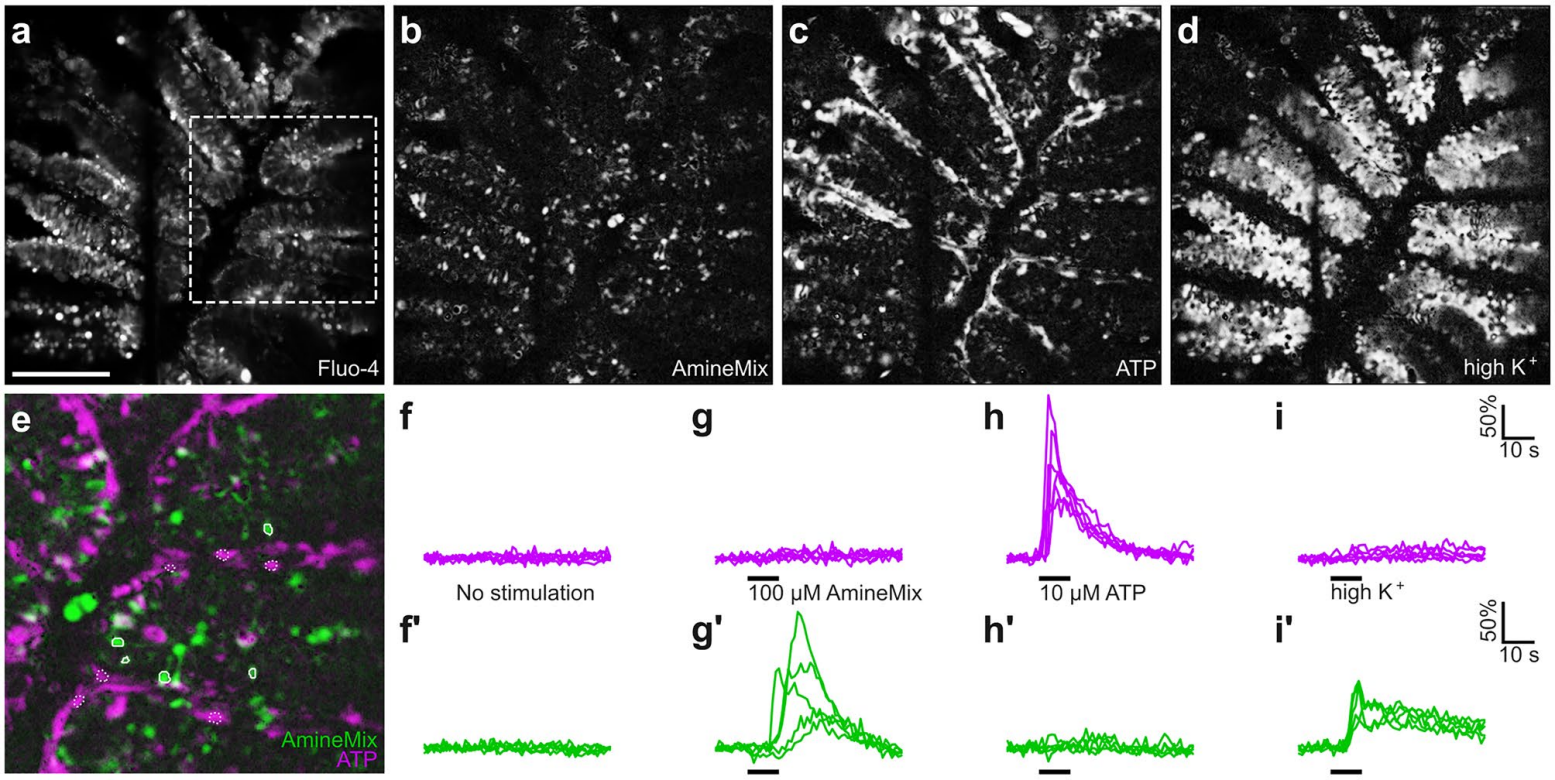
Odor-induced activity for two stimuli classes, amines, and nucleotides. Fluo4-loaded olfactory epithelium slice (**a**), fluorescence is averaged over complete experiment, dashed box is shown magnified in (**e**). Correlation maps for similarity in fluorescence changes upon stimulation (**b**–**d**). The degree of correlation of fluorescence changes after stimulation in neighboring pixels is shown in grey scale (**b**–**d**). The slice is stimulated with 100 μM amine mixture in ACSF (**b**), 10 μM ATP in ACSF (**c**), and high K^+^(**d**). **E** Overlay of correlation maps for amine mixture (green) and ATP (magenta) responses. ROIs for quantitative evaluation are shown with solid (amine mixture) or dashed (ATP) enclosure (**e**). Note that amines and ATP activate different subsets of cells in the olfactory epithelium. Traces for cells identified as ATP-responsive (**f**, **g**, **h**, **i**, magenta) and as amine-responsive (**f'**, **g'**, **h'**, **i'**, green). Note that only amine-responsive cells respond to high K^+^

To image odor-induced neuronal activity, olfactory epithelia of adult zebrafish were dissected, sliced horizontally, and incubated with the calcium-sensitive dye Fluo4-AM (Fig. 1), cf. (Weth et al. 1996, Fig. 1 for three-dimensional morphology). Stimuli were given as 10 s pulse. Cells reacting to stimuli (amine mixture and ATP) were identified in pixel correlation maps, and relative fluorescence changes (∆*F*/*F*_0_) were determined for the corresponding regions of interest (ROIs) over time (Fig. 2). Generally, signals could be replicated at least twice, albeit with small differences in the time course of the response (Fig. S1). Artificial cerebrospinal fluid (ACSF) was used as negative control (Fig. 2f, f') and high K^+^ was employed to ascertain the neuronal nature of the responsive cell (Fig. 2i'). Non-neuronal basal cells show no responses to high K^+^ (Fig. 2i).

### Amines constitute a major odor class for zebrafish

**Fig. 3:**
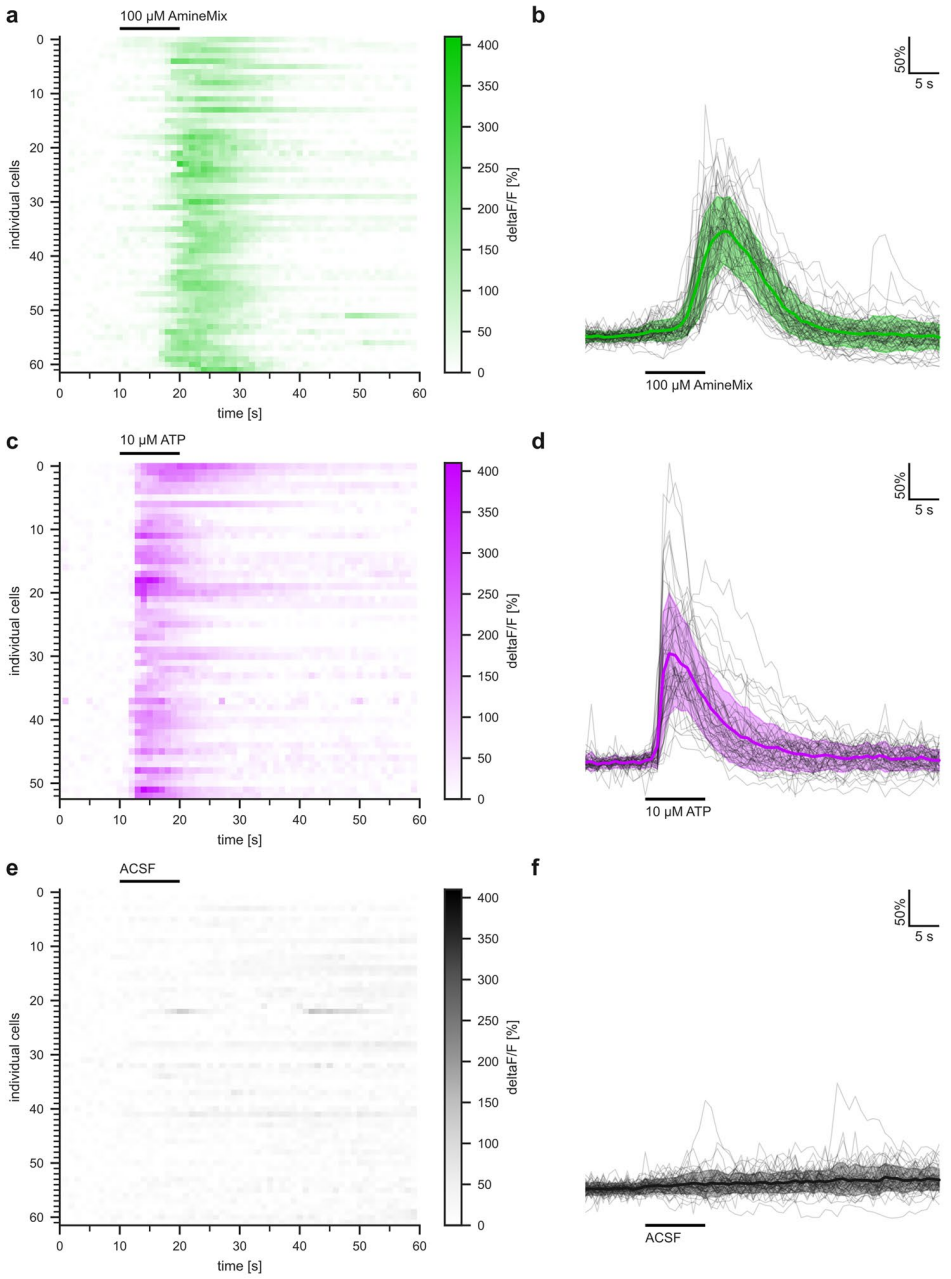
Calcium response kinetics for amines differ from those for ATP. Panels (**a**, **c**, **e**) heat maps represent color-coded changes in fluorescence over time in response to an odor pulse (black bar) for individual cells, number of cells as indicated. The respective color scales are shown to the right of the heat maps. Green colors, amine stimulus; magenta colors, ATP stimulus, concentrations as indicated. Panels (**b**, **d**, **f**) single cell (black curves) and averaged calcium response profiles (mean+/− SD, thick colored line and lightly colored area, respectively). 62 amine-responsive ROIs (**a**, **b**), 53 ATP-responsive ROIs (**c**, **d**), and 62 ACSF responses for the amine-responsive ROIs (**e**, **f**, negative control), all results are from the same slice

In preliminary experiments we saw very little responses to amino acids (composition of mix see Manzini and Schild 2004), which are known odorants for fishes (Hara 1975; Friedrich and Korsching 1997). In contrast, frequent and strong responses were observed for the amine mixture and for ATP (Figs. 2 and 3). The neuronal subpopulations mediating amino acid, amine, and nucleotide responses are all supposed to be different (microvillous, ciliated, and pear neurons, respectively, cf. Koide et al. 2009; Pacifico et al. 2012; Dieris et al. 2017; Wakisaka et al. 2017) and may exhibit differential dye uptake and/or processing (cf. Friedrich and Korsching 1997; Manzini and Schild 2003).

The amine mixture used as stimulus consisted of primary, secondary, and tertiary amines, and included aliphatic as well as aromatic amines. This formulation was designed to ensure a comprehensive representation of amine-responsive cells. For each amine 100 µM concentration was employed, which is close to saturation for the presumed receptors (TAARs, Hussain et al. 2013; Li et al. 2015) and thus should ensure a robust and comprehensive response. Amine responses were generally frequent; sometimes only few, but often over 100 cells were activated by amines in a single optical layer. Responses to amines without exception showed a later onset compared to ATP responses measured in the same experiment, and were generally slightly smaller in intensity (Figs. 2 and 3). Neither amine- nor ATP-responding cells showed any signals with the mock stimulus, ACSF. Amine-responsive cells generally did not react to ATP and vice versa; rare exceptions can not be differentiated unambiguously from signals in two superimposed cells. Next, we quantified the response kinetics of amine-responsive cells and compared them to the ATP-responding cells.

### Kinetics of amine responses are characteristically different from ATP responses

**Fig. 4:**
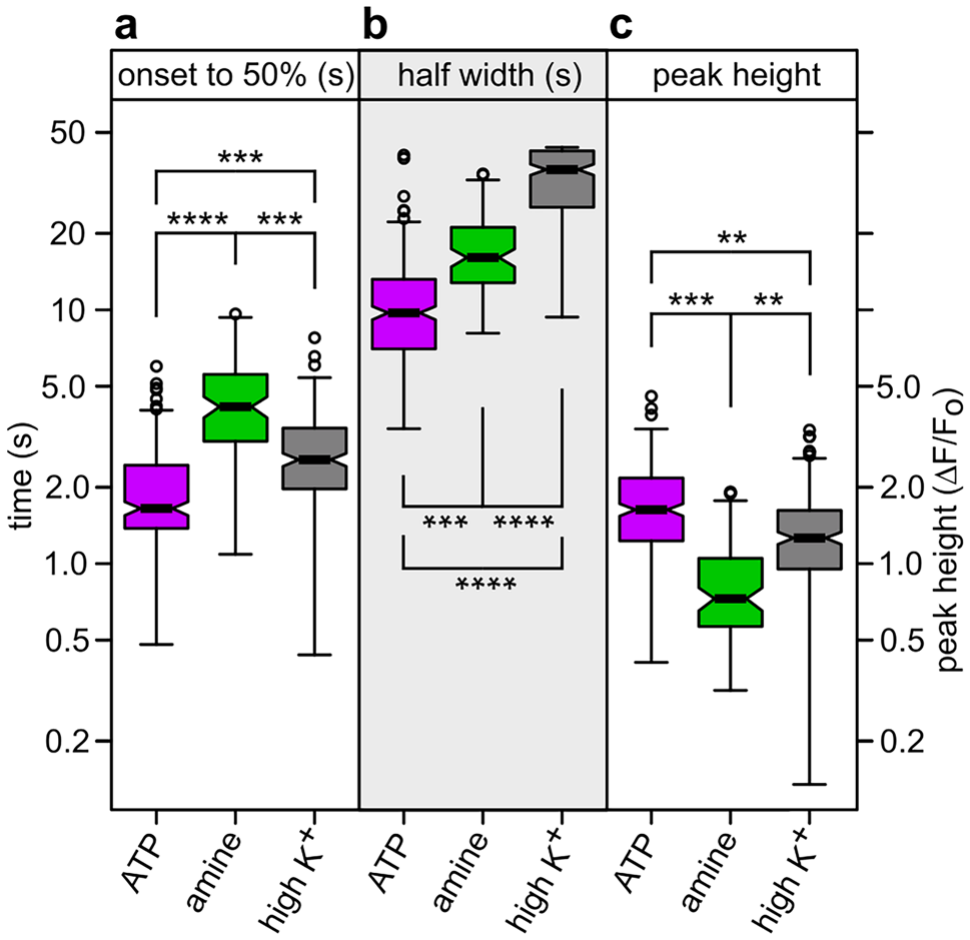
Statistical analysis shows amine responses significantly different from ATP responses. Calcium responses were measured for cells responding to the amine-mix (103 cells) and to ATP (269 cells). No overlap in cell populations was seen. Additionally, responses to high K^+^ (225 cells) were measured at the end of an experiment. Three kinetic parameters were quantified: Onset time, measured from onset of response to 50% of maximal peak height (**a**, time to 50%), peak width at half-maximal height (**b**, half width), and maximal peak height (**c**, peak height), expressed as ∆*F*/*F*_0_. Distributions are shown as box plots, notches represent 95% confidence interval, outliers are shown as circles. Significance was estimated with two-tailed *t*-test, **p < 10^–10^, ***p < 10^–15^; ****p < 10^–30^. Note that all three response characteristics are highly significantly different for amine vs. ATP responses

For quantification we measured three parameters: an onset time, defined as time from onset of response to 50% peak height, the width of the peaks at 50% peak value, and the maximal peak height. The onset time includes any signal transduction processes before the readout parameter (fluorescence of the calcium-sensitive dye). Both time to half-maximal peak and halfwidth of the peak are significantly larger for amine-responding cells than for the ATP-responsive cells (Fig. 4a, b, *p* < 10^–30^ and *p* < 10^–15^, respectively), suggesting different modes of signal transduction in these two cell populations. ATP receptors include ion channels (P2X receptors), and those can respond faster than G protein-coupled receptors (TAARs) that require additional steps of signal transduction. The peak intensity of amine-responsive cells is somewhat smaller than for ATP-responsive cells (Fig. 4c, *p* < 10^–15^). For comparison we have also quantified responses to high K^+^ (Fig. 4). The onset time lies below the value for amine stimuli, which is expected: high K^+^-induced depolarization can directly open voltage-sensitive calcium channels, whereas amine responses require additional steps of signal transduction before channels can open. The high K^+^ responses are not fully reversible in the investigated time window, which results in larger halfwidths compared to those for responses to ATP and amines.

### Laminar distribution of amine-responding cells is highly similar to that of ciliated OSNs and significantly different from microvillous OSN and basal cells

**Fig. 5:**
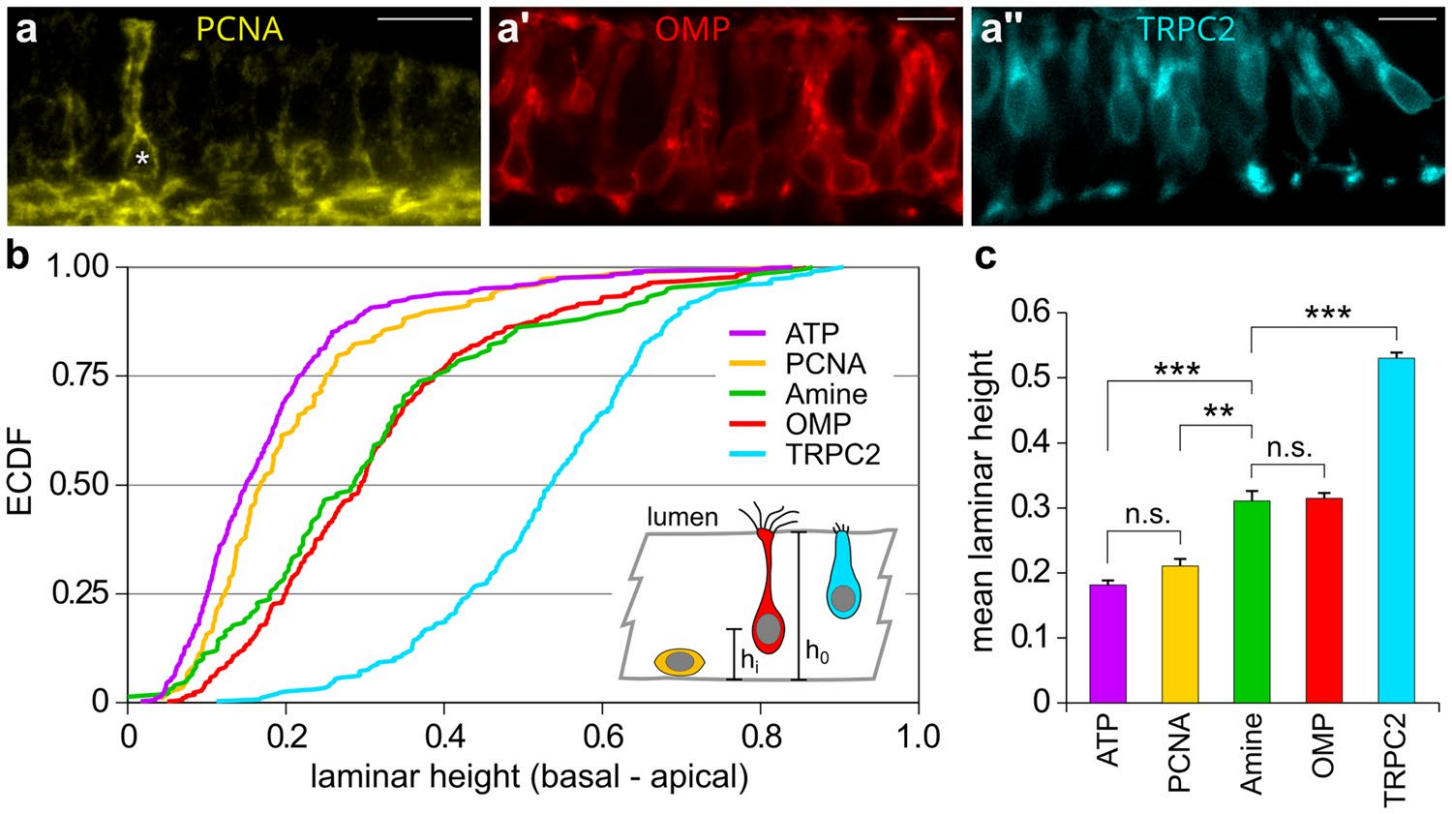
Spatial distribution of amine-responding cells equals that of OMP-positive cells. Representative half-lamella segments of olfactory epithelium (**a**, **a'**, **a''**), basal is down, apical (toward lumen) is up. PCNA, IHC staining (**a**); OMP (**a'**) and TRPC2 (**a''**), transgene labeling. PCNA and TRPC2 signal are depicted in false color. Scale bars 10 μm. Laminar height within the lamella (**b**) is determined as the distance from the basal lamina to cell soma center (*h*_i_) divided by the total laminar height h_0_ at that position and is depicted as empirical cumulative distribution function for five cell populations: ATP, ATP-responsive cells in calcium imaging; PCNA, PCNA-immunoreactive cells; Amine, amine-responsive cells in calcium imaging; OMP, OMP-transgene; TRPC2, TRPC2 transgene. Note that occasionally PCNA+ cells with neuronal morphology are observed (**a**, asterisk), these are most likely immature neurons that still retain some PCNA protein. The inset in (**b**) shows the schematical cell shapes for progenitor cells (PCNA+), ciliated (OMP+), and microvillous (TRPC2+) neurons. Mean laminar height (**c**) for the five cell populations depicted in (**b**), color code as for (**b**). Error bars depict SEM. Amine responses are distinctly and significantly different from both PCNA and TRPC2-expressing cells, but appear identical to OMP-expressing cells. **p < 10^–7^; ***p < 10^–15^

Next we investigated the cell type of amine-responsive cells. They may be expected to comprise ciliated OSNs expressing *taar* genes from the large zebrafish TAAR family (112 genes; Hussain et al. 2009). In zebrafish, ciliated neuron somata occupy an intermediate layer between basal nonneuronal cells and apical microvillous receptor neurons (Ahuja et al. 2014). We quantified laminar height for amine-responsive and ATP-responsive cells and compared these distributions with those of marker genes. We used an antibody for proliferating cell nuclear antigen, PCNA, to label basal cells (Fig. 5a), a transgenic line expressing red fluorescent protein, RFP, under control of the OMP promoter (olfactory marker protein) to label ciliated neurons (Fig. 5a'), and a transgenic line expressing venus under control of the TRPC2 promoter (transient receptor potential channel C2) to label microvillous neurons (Fig. 5a'') (Ino and Chiba 2000; Sato et al. 2005).

The laminar height distribution of amine-responding cells is extremely similar to that of ciliated neurons marked by OMP expression and significantly different from all other distributions (Fig. 5b, c), suggesting that amine responses occur in ciliated neurons. Notably, the distribution of PCNA-expressing cells closely resembles that of ATP-responding cells (Fig. 5b, c), suggesting that ATP responses originate mostly from basal nonneuronal cells, consistent with earlier findings (Demirler et al. 2020). As expected, no calcium responses align with the distribution of microvillous neurons, as visualized by TRPC2 expression (Fig. 5b, c).

## Discussion

Zebrafish is a common vertebrate model system to study olfaction (Miyasaka et al. 2013; Korsching 2020). While the olfactory receptor repertoire has been analyzed in some detail (Korsching 2020), fewer physiological studies have analyzed odor-induced activity in olfactory brain regions (Kermen et al. 2013). To the best of our knowledge, no studies have examined odor-induced activity in the sensory surface itself, apart from earlier electroolfactogram studies (Rolen et al. 2003; Michel et al. 2003) or histological detection of neuronal activity markers pERK and cfos in some specialized cases (Hussain et al. 2013; Yabuki et al. 2016; Dieris et al. 2017; Wakisaka et al. 2017). Here we have established an ex vivo calcium imaging technique tailored for zebrafish olfactory epithelium. Using this technique, we reveal amines as major odor class in aquatic vertebrates. The response characteristics of amine-responsive cells are clearly distinct from those of the nonneuronal ATP-responding cells—the latter are consistent with earlier reports (Demirler et al. 2020). The laminar position of amine-responsive cells in the olfactory epithelium suggests them to be ciliated OSNs, in accordance with expectations derived from a specialized case (cadaverine-responsive OSNs; Dieris et al. 2017).

A major axonal destination for amine-responsive OSNs in the olfactory bulb appears to be the dorsolateral cluster, encompassing approximately 50 distinguishable glomeruli (Baier and Korsching 1994; Braubach et al. 2012; Dieris et al. 2017). Within this cluster, TAAR13c-expressing neurons specifically innervate one glomerulus (Dieris et al. 2017). The taar gene family, the second-largest olfactory receptor family in zebrafish (Hussain et al. 2009), includes several members demonstrated to respond to diverse amines in heterologous expression experiments (Li et al. 2015; Sharma et al. 2016), akin to their mammalian counterparts (Dewan et al. 2013). These results, together with the functional characterisation reported here, are consistent with the hypothesis that the dorsolateral cluster may generally be innervated by TAAR-expressing neurons. The number of glomeruli is in reasonable agreement with the number of taar genes (50 vs. 112, Hussain et al. 2009; Braubach et al. 2012), considering that closely related olfactory receptor genes may be co-expressed (Sato et al. 2007), and that the dorsolateral cluster is very dense, (cf. Baier and Korsching 1994), which might lead to underestimation of the glomerular number. Similarly, in mammals glomeruli innervated by TAAR-expressing neurons form an anatomically segregated subdomain of the olfactory bulb (Pacifico et al. 2012). At the behavioral level, some amines have been shown to elicit aversive reaction, and may serve to signal predators (mammals, Dewan et al. 2013) or generalized danger (zebrafish, Hussain et al. 2013).

Processing olfactory information comprises many steps, from detection of amines by their cognate receptors to finally eliciting behavior. Many of these steps had been described already. Our results for the activation of OSNs in the sensory surface form the last missing link establishing amines as a relevant odor class for teleost fish.

## Materials and methods

### Animal and tissue handling

Animal housing and maintenance was licensed by the office for environment and consumer protection of the city of Cologne, Germany. Zebrafish used in this study were raised in the local fish facility at 28 °C with a 14/10 photoperiod.

Adult zebrafish (6–18 months) were euthanized with MS-222, and decapitated. Olfactory epithelia were dissected in ACSF and embedded in 2.5% low-melting agarose. After hardening the olfactory epithelium was cut horizontally by vibratome (Leica VT1200S) to obtain two planar surfaces suitable for imaging. The agarose slices were incubated with the calcium-sensitive dye Fluo4-AM (50 μg/ml in ACSF) in the dark for 30 min at RT as described (Hassenklöver et al. 2009). The quality of the preparation was assessed by vigorous cilia movement in the non-sensory regions of the epithelium.

### Odor stimuli, calcium imaging, and data analysis

To obtain representative responses to amine stimuli, a mix of thirteen different primary, secondary, and tertiary amines (2-phenylethylamine, tyramine, butylamine, cyclohexylamine, hexylamine, 3-methylbutylamine, N,N-dimethylethylamine, 2-methylbutylamine, 1-formylpiperidine, 2-methylpiperidine, N-ethylcyclohexylamine, 1-ethylpiperidine, piperidine) was employed as described (Gliem et al. 2013). Individual concentrations were 100 µM. ATP was used at 10–100 µM. No stimulus control consisted of a mock stimulus with ACSF. The high K^+^ stimulus (131 mM, obtained by exchanging sodium and potassium concentrations in the ACSF solution) was given at the end of the experiments.

Calcium imaging was performed essentially as described (Hassenklöver et al. 2009). In short, agarose-embedded tissue slices were placed in an imaging chamber under constant ACSF flow. Stimuli (10–100 µM) were added into the flow, resulting in correspondingly diluted final concentrations. Images (512 × 512 pixel) were taken with a confocal microscope (Zeiss LSM 780, Argon Laser, excitation at 488 nm/emission at > 495 nm) at 1 Hz. Stimulus was given 10 s after onset of recording for another 10 s, total recording time per stimulus was 60 s. Interstimulus interval was at least 3 min. The optical slice thickness was set to be smaller than one cell layer to (nearly) exclude superposition of signals from different cells.

Image analysis was performed using custom scripts written in MATLAB (MathWorks, USA) as described previously (Junek et al. 2009; Dieris 2018). In short, a pixel correlation map was created to facilitate the selection of regions of interest (ROIs). Correlated changes in intensity after stimulation serve to delineate responsive cells. Fluorescence changes for individual ROIs were then plotted as Δ*F*/*F*_0_ with *F*_0_ being the average fluorescence from the first 10 images. Averaged data from multiple ROIs were plotted as mean Δ*F*/*F*_0_ ± SD.

### Measurement and analysis of spatial coordinates

The laminar height of cell somata within the lamella is significantly different for different cell populations and can thus serve to characterize the responsive cell population. Laminar height was determined as described previously (Kowatschew et al. 2022) for cells with odor responses and for PCNA-, OMP- and TRPC2-expressing cells (the latter two in transgenic reporter lines OMP:lynRFP and TRPC2:gap-Venus (Sato et al. 2005)). In short, the distance from the basal lamina to cell soma center (h_i_) and the total laminar height h_0_ at that position were determined, and the ratio h_i_/h_0_ was plotted as empirical cumulative distribution function (ECDF, Feller 1967; Wilk and Gnanadesikan 1968).

### Immunohistochemical staining

Tissues were fixed in 4% PFA for 1 h at room temperature before dissection of the olfactory epithelia, which were then embedded in TissueTek (Sakura). Ten-micrometer cryosections were prepared, dried, re-hydrated, and blocked in 3% BSA-PBST for 1 h. The anti-PCNA primary antibody (mouse anti-PCNA, Merck) was diluted 1:250 in blocking solution and incubated overnight. After 3 washes, secondary antibody was applied at 1:250 dilution for 3 h at room temperature. Sections were mounted with VECTASHIELD^®^ mounting medium (Vector Laboratories). Images were obtained with a Keyence BZ-9000 wide field fluorescence microscope (Keyence, Japan).

### Supplementary information

Below is the link to the electronic supplementary material.

Supplementary file1 (PDF 119 KB)

## Data Availability

All data are included in the article and its supplementary material.

## References

[CR1] Ahuja G, Bozorg Nia S, Zapilko V (2014). Kappe neurons, a novel population of olfactory sensory neurons. Sci Rep.

[CR2] Baier H, Korsching S (1994) Olfactory glomeruli in the zebrafish form an invariant pattern and are identifiable across animals. J Neurosci 14:219–230 10.1523/JNEUROSCI.14-01-00219.1994 PMC6576838 8283233

[CR3] Braubach OR, Fine A, Croll RP (2012). Distribution and functional organization of glomeruli in the olfactory bulbs of zebrafish (*Danio rerio*). J Comp Neurol.

[CR4] Czesnik D, Kuduz J, Schild D, Manzini I (2006). ATP activates both receptor and sustentacular supporting cells in the olfactory epithelium of *Xenopus laevis* tadpoles. Eur J Neurosci.

[CR5] DeMaria S, Berke AP, Name EV (2013). Role of a ubiquitously expressed receptor in the vertebrate olfactory system. J Neurosci.

[CR6] Demirler MC, Sakizli U, Bali B (2020). Purinergic signalling selectively modulates maintenance but not repair neurogenesis in the zebrafish olfactory epithelium. FEBS J.

[CR7] Dewan A, Pacifico R, Zhan R (2013). Non-redundant coding of aversive odours in the main olfactory pathway. Nature.

[CR8] Dieris M (2018) Amine detection in aquatic organisms: receptor evolution, neuronal circuits and behavior in the model organism zebrafish. PhD Thesis, University of Cologne

[CR9] Dieris M, Ahuja G, Krishna V, Korsching SI (2017). A single identified glomerulus in the zebrafish olfactory bulb carries the high-affinity response to death-associated odor cadaverine. Sci Rep.

[CR10] Feller W (1967) An introduction to probability theory and its applications, 3rd edn. Wiley, New York

[CR11] Friedrich RW, Korsching SI (1997). Combinatorial and chemotopic odorant coding in the zebrafish olfactory bulb visualized by optical imaging. Neuron.

[CR12] Friedrich RW, Korsching SI (1998) Chemotopic, combinatorial, and noncombinatorial odorant representations in the olfactory bulb revealed using a voltage-sensitive axon tracer. J Neurosci 18:9977–9988 10.1523/JNEUROSCI.18-23-09977.1998 PMC6793301 9822753

[CR13] Gliem S, Syed AS, Sansone A (2013). Bimodal processing of olfactory information in an amphibian nose: odor responses segregate into a medial and a lateral stream. Cell Mol Life Sci.

[CR14] Hara TJ (1975). Olfaction in fish. Prog Neurobiol.

[CR15] Hassenklöver T, Schwartz P, Schild D, Manzini I (2009) Purinergic signaling regulates cell proliferation of olfactory epithelium progenitors. Stem Cells (Dayton, Ohio) 27:2022–2031. 10.1002/stem.126 10.1002/stem.126 19544419

[CR16] Hussain A, Saraiva LR, Ferrero DM (2013). High-affinity olfactory receptor for the death-associated odor cadaverine. Proc Natl Acad Sci USA.

[CR17] Hussain A, Saraiva LR, Korsching SI (2009). Positive Darwinian selection and the birth of an olfactory receptor clade in teleosts. Proc Natl Acad Sci USA.

[CR18] Ino H, Chiba T (2000). Expression of proliferating cell nuclear antigen (PCNA) in the adult and developing mouse nervous system. Brain Res Mol Brain Res.

[CR19] Junek S, Chen T-W, Alevra M, Schild D (2009). Activity correlation imaging: visualizing function and structure of neuronal populations. Biophys J.

[CR20] Kermen F, Franco LM, Wyatt C, Yaksi E (2013) Neural circuits mediating olfactory-driven behavior in fish. Front Neural Circuits 7:62. 10.3389/fncir.2013.00062 10.3389/fncir.2013.00062 PMC3622886 23596397

[CR21] Koide T, Miyasaka N, Morimoto K (2009). Olfactory neural circuitry for attraction to amino acids revealed by transposon-mediated gene trap approach in zebrafish. Proc Natl Acad Sci.

[CR22] Korsching SI (2020) Taste and smell in zebrafish. Fritzsch B (ed) and Meyerhof W (vol Editor). Academic Press, Elsevier, pp 466–492

[CR23] Kowatschew D, Bozorg Nia S, Hassan S (2022). Spatial organization of olfactory receptor gene choice in the complete V1R-related ORA family of zebrafish. Sci Rep.

[CR24] Li Q, Tachie-Baffour Y, Liu Z et al (2015) Non-classical amine recognition evolved in a large clade of olfactory receptors. eLife 4:e10441. 10.7554/eLife.10441 10.7554/eLife.10441 PMC4695389 26519734

[CR25] Liberles SD, Buck LB (2006). A second class of chemosensory receptors in the olfactory epithelium. Nature.

[CR26] Manzini I, Schild D (2003). Multidrug resistance transporters in the olfactory receptor neurons of *Xenopus laevis* tadpoles. J Physiol.

[CR27] Manzini I, Schild D (2004). Classes and narrowing selectivity of olfactory receptor neurons of *Xenopus laevis* tadpoles. J Gen Physiol.

[CR28] Michel WC, Sanderson MJ, Olson JK, Lipschitz DL (2003). Evidence of a novel transduction pathway mediating detection of polyamines by the zebrafish olfactory system. J Exp Biol.

[CR29] Miyasaka N, Wanner AA, Li J (2013). Functional development of the olfactory system in zebrafish. Mech Dev.

[CR30] Mombaerts P (2004). Genes and ligands for odorant, vomeronasal and taste receptors. Nat Rev Neurosci.

[CR31] Mombaerts P (2006). Axonal wiring in the mouse olfactory system. Annu Rev Cell Dev Biol.

[CR32] Pacifico R, Dewan A, Cawley D (2012). An olfactory subsystem that mediates high-sensitivity detection of volatile amines. Cell Rep.

[CR33] Rolen SH, Sorensen PW, Mattson D, Caprio J (2003). Polyamines as olfactory stimuli in the goldfish *Carassius auratus*. J Exp Biol.

[CR34] Sato Y, Miyasaka N, Yoshihara Y (2005) Mutually exclusive glomerular innervation by two distinct types of olfactory sensory neurons revealed in transgenic zebrafish. J Neurosci 25:4889–4897. 10.1523/JNEUROSCI.0679-05.2005 10.1523/JNEUROSCI.0679-05.2005 PMC6724860 15901770

[CR35] Sato Y, Miyasaka N, Yoshihara Y (2007) Hierarchical regulation of odorant receptor gene choice and subsequent axonal projection of olfactory sensory neurons in zebrafish. J Neurosci 27:1606–1615. 10.1523/JNEUROSCI.4218-06.2007 10.1523/JNEUROSCI.4218-06.2007 PMC6673750 17301169

[CR36] Sharma K, Ahuja G, Hussain A (2016). Elimination of a ligand gating site generates a supersensitive olfactory receptor. Sci Rep.

[CR37] Stacey N, Chojnacki A, Narayanan A (2003). Hormonally derived sex pheromones in fish: exogenous cues and signals from gonad to brain. Can J Physiol Pharmacol.

[CR38] Wakisaka N, Miyasaka N, Koide T et al (2017) An adenosine receptor for olfaction in fish. Curr Biol 27:1437–1447.e4. 10.1016/j.cub.2017.04.014 10.1016/j.cub.2017.04.014 28502661

[CR39] Weth F, Nadler W, Korsching S (1996). Nested expression domains for odorant receptors in zebrafish olfactory epithelium. Proc Natl Acad Sci USA.

[CR40] Wilk MB, Gnanadesikan R (1968). Probability plotting methods for the analysis of data. Biometrika.

[CR41] Yabuki Y, Koide T, Miyasaka N (2016). Olfactory receptor for prostaglandin F2α mediates male fish courtship behavior. Nat Neurosci.

