# Supplementary material for: Calcium imaging of adult olfactory epithelium reveals amines as important odor class in fish
Source: Cell Tissue Res. 2024 Feb 13;396(1):95–102. doi: 10.1007/s00441-024-03859-w (PMC10997700; doi:10.1007/s00441-024-03859-w)
Supplement: Supplementary file 1 — Supplementary file1 (PDF 119 KB) [file 441_2024_3859_MOESM1_ESM.pdf]

Supplementary Figure 1

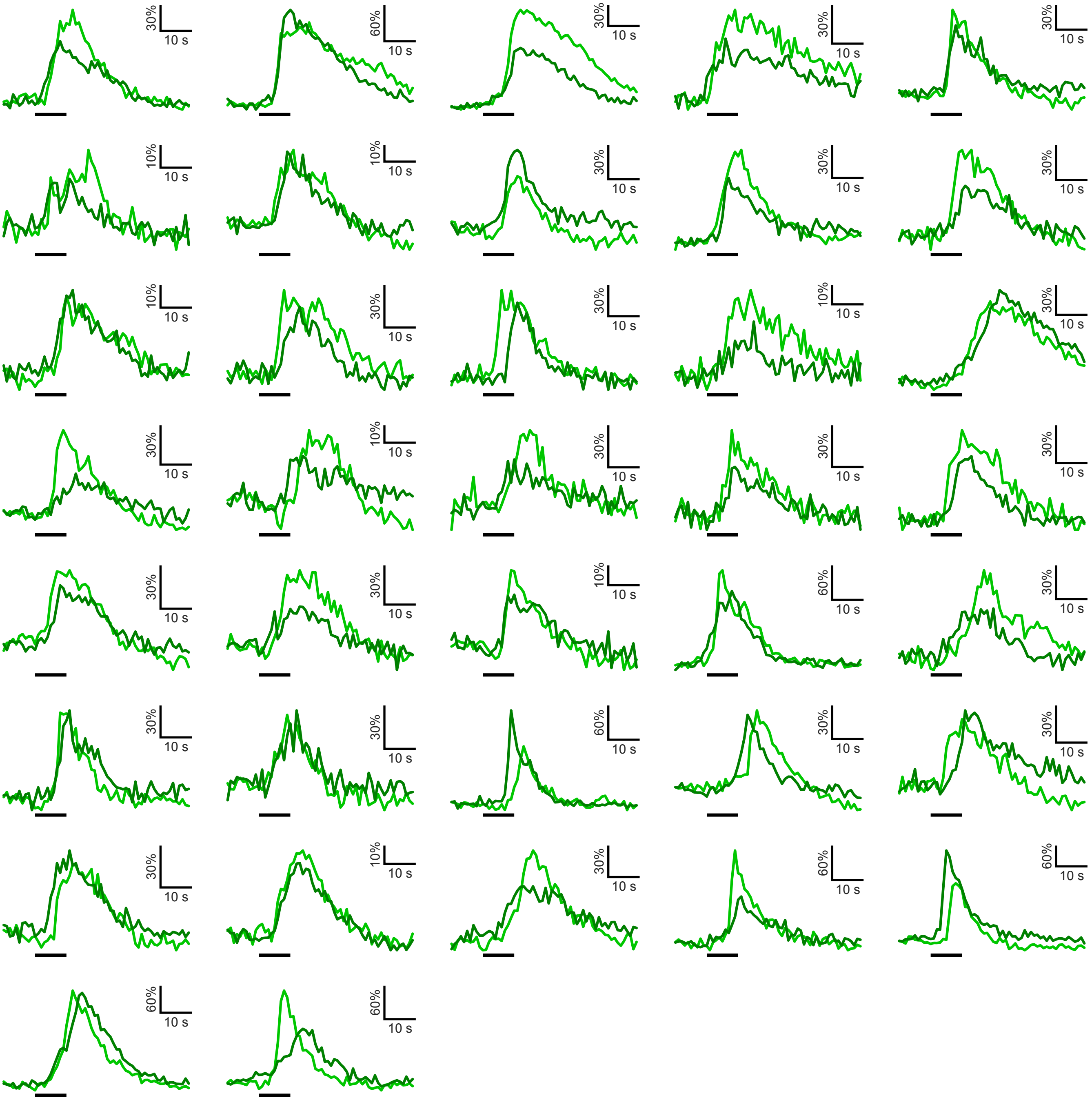

Repeat stimulation shows the reproducibility of the amine responses

ΔF/F traces from individual cells in a Fluo4-loaded olfactory epithelium slice of adult zebrafish after stimulation with 100 μM amine mixture in ACSF. Dark green trace, first stimulation; light green trace, repeat stimulation. Note the reproducibility of the responses.
